# The Effect of *Teucrium Polium* Hney on the Wound Healing and Tensile Strength in Rat

**Published:** 2011

**Authors:** Ali Mohammad Alizadeh, Hamid Sohanaki, Mahmod Khaniki, Mohammad Ali Mohaghgheghi, Giti Ghmami, Maryamsadat Mosavi

**Affiliations:** 1 *Cancer Research Centre of Institute Cancer, Tehran University of Medical Sciences, Tehran, Iran*; 2 *Department of Physiology, School of Medicine, Tehran University of Medical Sciences, Tehran, Iran*; 3 *Department of Pathology, School of Medicine, Tehran University of Medical Sciences, Tehran, Iran*; 4 *Department of Pharmacology, School of Medicine, Tehran University of Medical Sciences, Tehran, Iran*

**Keywords:** Honey, Rat, Teucrium polium, Wound incision, Wound excision

## Abstract

**Objective(s):**

Wound healing represents a dynamic physiological process influenced by many factors. The aim of the present study was to evaluate the effects of *Teucrium polium* honey on the wound healing and tensile strength in rat.

**Materials and Methods:**

Thirty-six Sprague-Dawley rats were randomly divided into four equal (n= 9) treatment and control groups. Two full-thickness wounds were made over the dorsal thoracic region according to the incision and excision models. Animals were treated with topical *Teucrium polium* honey twice a day post surgery until complete healing was achieved. Histopathology and tensiometry were then studied.

**Results:**

The wound healing process occurred faster in the incision model than excision ones (*P*< 0.05). *Teucrium polium* honey promoted wound contraction, closure time and tensile strength (*P*< 0.05). Histopathological study also showed relative epithelial proliferation, improved angiogenesis granulation, and fibrous connective tissue in *Teucrium polium* honey treated animals.

**Conclusion:**

The present study demonstrates that *Teucrium polium *honey can accelerate wound healing as well as tensile strength in rat skin wounds.

## Introduction

Wounds are still a major problem in developing countries, often imposing severe complications and high cost for therapy. Wound healing is a set of coordinated responses to tissue injury. It represents a dynamic physiological process initiated and influenced by many factors seen in homeostasis, inflammation, proliferation and remodeling phases (1). Wound care is often complex, frequently time-consuming, sometimes confusing and nearly always expensive. Plant products are widely used as medicaments for wound healing in many clinical situations due to their cost effectiveness, widespread availability, non-toxicity, ease of use, fewer side effects, and patient compliance. Botanical honey with potential effects on wound healing has been demonstrated repeatedly. It has long been utilized for skin injuries and other medical conditions. However, it can be used in unsterile conditions, as it already possesses a bactericidal property (2-5). 

Several studies suggest that honey reduces inflammation, swelling, pain and promotes debridement, granulation, epithelialization and healing with minimal scarring (6,7). The antibacterial activity of honey is well-documented, along with the publications of numerous case studies and trials reporting improved healing rates following honey application (8). Honey is easy to use and readily acceptable by the patients.

*Teucrium polium* is a medicinal plant, several species of which have been used over 2000 years in traditional medicine. Its white or pale cream colored flowers appear in April until August. The plant is usually found in most parts of Mediterranean and Irano-Turaian regions (9). *Teucrium polium* is widely used in inflammatory conditions, rheumatism and ulcers (10,11). Therefore, we decided to examine the potential effects of *Teucrium polium* honey on wound healing and tensile strength in the animal model.

## Materials and Methods

***Honey***

South Khorasan originated *Teucrium polium* honey provided by the Scientific Herbal and Bestial Research Institute (Hesarak St, , ) was used. It was stored in a dark glass container at room temperature. Samples were taken to investigate antimicrobial properties against control solution with the same sugar content of natural honey.

***Animals***

Thirty six male Sprague–Dawley rats (250-300 g) were maintained in the animal quarters under standardized conditions. They received laboratory chow and water *ad libitum*. Animals were randomly assigned into four equal (n= 9) groups of two excision and incision and their respective control groups. No topical or systematic therapy was used except for Teucrium Polium honey which was applied twice a day post surgery until complete healing of the wound. Investigation procedures were approved by the animal ethics committee of Tehran University of Medical Sciences. 

***Surgical preparation***

The preparation used in the present study was previously described (12). Briefly, the animals were anaesthetized by a mixture of ketamine (50 mg/kg i.p.) and xylazine (5 mg/kg i.p.). They were shaved on the back and stabbed with two full-thickness incision (2 cm in length) and/or excision (1 cm in diameter) wounds using sterile surgical scalpels on the dorsal thoracic region (1 cm lateral to the vertebral column, 5 cm posterior to the intraaural line) in anaesthetized rat. Animals were then kept in separate cages. Treatment started with unprocessed *Teucrium polium* honey (2 g) twice a day from day 0 until complete healing of the wound (day 18).

***Wound area measurement***

The progressive changes in the wound length and area were measured and recorded on a graph paper every 3 day until complete wound healing. Wound contraction was expressed as a reduction in percentage of the original wound size (13). The animals were anesthetized by ether and then the shape of the wound was drawn on transparent film by a special marker. The wound area was accurately measured and the percentage of healing was calculated by negatoscope and Video Image Analyzer software according to the following formula on different days (12). 

The percentage of wound healing on day X=100 - wound length or area / wound length×100. 

***Histopathological study***

Histopathological study was done on 4th, 8th, and 12th post operation days (three rats in each group) using healing markers like keratinization, epithelization, collagenation, fibrosis and neovascularisation. Sections were taken and immediately fixed with 10% formalin solution, dehydrated with 90% ethanol and embedded in paraffin. Then, they were cut into thin slices and stained with Haemotoxyline-Eosin (H & E) and observed under a light microscope. Wound changes were pathologically assessed and reported.

***Tensiometry study***

After healing completion, animals were killed by chloroform inhalation (six rats in each group). Dorsal skin was excised at the deep fascia and put immediately in normal saline to prevent drying (12). Then, a narrow strip (4 cm in length and 3 cm in width) was attached to tensiometer holders (Tensiometry, Co. ). The tissue stress (maximum force tensile leading to skin rupture), tissue strain (tissue length under maximum tension) and fracture energy (stress-strain AUC) were evaluated. 

***Statistical analysis***

Data were expressed as mean±SEM statistical differences between respective treated and their control groups were analyzed by student’s t-test. Within group analysis was performed with repeated measures one–way analysis of variance (ANOVA). Statistical significance was defined as *P*< 0.05. 

## Results

***Excision and incision wounds***

Mean healed wound area percentage on days 6, 9, 12 and 15 was significantly increased in the incision wound group compared to the respective controls (*P*< 0.05). In the excision wound model, it has also been increased on days 6, 9, 12 and 15 compared to its control. Mean healed wound area percentage on days 6, 9 and 12 was significantly increased in the excision wound group compared to the respective controls (*P*< 0.05) (Table 1). Also mean healed wound area percentage on days 6 and 9 was significantly increased in the incision wound in comparison with the excision wound group (student’s t-test, *P*< 0.05) 

***Histopathology***

**Figure 1.**
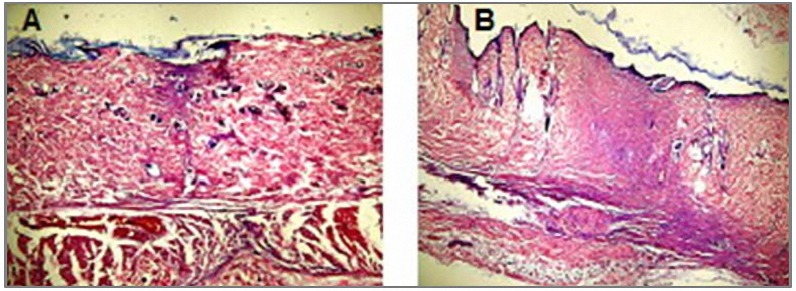
A feature of incision wound on day 12. A: Treated group, B: Control group (X250, H & E).

**Figure 2.**
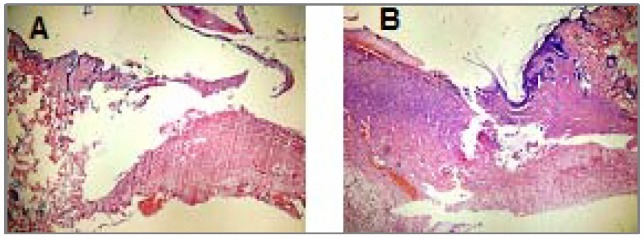
A feature of excision wound on day 12. A: Treated group, B: Control group (X250, H & E).

Light microscopic study of H & E and Masson’s trichrome stained tissues on days 4, 8 and 12 showed overall faster healing in treated groups (*P*< 0.05). Re-epithelialization, granulation tissue formation, collagen arrangement, fibrinoleukocytic exudates severity, vasculature hyperemia or congestion, dermis organization and epidermal appendage reappearance of the injured area were all improved in the treated groups (Table 2, Figures 1, 2).

**Table 1.** The percentage of wound healing area in the incision and the excision wounds on different days

Days	0	3	6	9	12	15	18

Groups							
IC	0	10±1.4	31.7±0.6	47.6±3.1	73±2.6	100	
IT	0	18.6±4	^a^ 61 ±3.3	^a ^ 93±9.4	^c^ 98±2	100	
EC	0	10±1.4	27.1±2	53.7±2.7	70.1±3.8	88±4	100
ET	0	16.3±1.5	^c^ 48.1±4	^b^ 70.3±3	^c^ 94±1	100	

Data are expressed as mean±SEM.^ a^*P*< 0.05, ^b^*P*< 0.01 and ^c^*P*< 0.001 vs. control group. IC: The incision wound of control group, IT: The incision wound of treated group, EC: The excision wound of control group, ET: The excision wound of treated group.

**Table 2.** Histopathological parameters in the incision and the excision wounds on different days

ParameterSample	Epidermization	Granulation tissue	Collagenization	Fibrinoleukocytic inflammation	Hyperemia/congestion & hemorrhage	Organization of dermis	Presence of skin adnexa
EC_1_	0	±	0	++	++	0	0
EC_2_	±	++	±	+	+	±	0
EC_3_	+	+	+	+	++	++	±
ET_1_	0	±	±	++	++	±	0
ET_2_	+	++	+	±	±	+	0
ET_3_	++	±	++	0	0	++	+
IC_1_	0	+	0	++	+	0	0
IC_2_	±	±	±	+	±	±	0
IC_3_	+	0	+	±	±	++	±
IT_1_	±	±	±	+	+	±	0
IT_2_	±	±	+	±	±	+	±
IT_3_	++	0	++	0	0	+++	++

Qualitative differences of histopathological parameters are depicted in details on different days: 0; nil, ±; slight, +; low, ++; high. IC; Incision wound control group, IT; Incision wound treated group, EC; Excision wound control group, ET; Excision wound treated group. Indices 1, 2 and 3 indicate days 4, 8, and 12, respectively.

**Figure 3.**
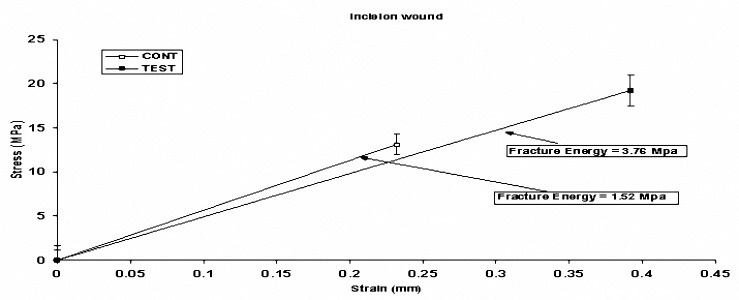
Schematic diagram of fracture energy in incision wound on day 18. Data are expressed as mean±SEM. Stress: Maximum force tensile leading to skin rupture, Strain: Tissue length under maximum tension, Fracture energy: Stress-strain AUC. *P*< 0.05 vs. control group.

**Figure 4.**
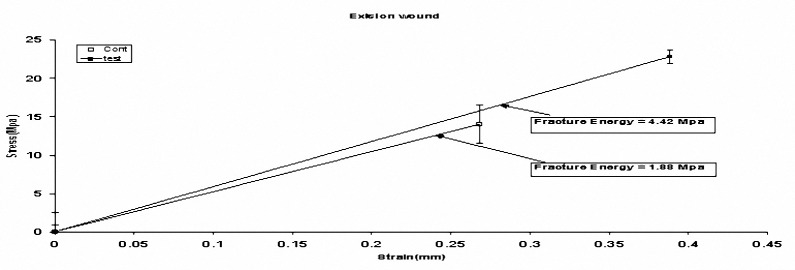
Schematic diagram of fracture energy in excision wound on day 18. Data are expressed as mean±SEM. Stress: Maximum force tensile leading to skin rupture, Strain: Tissue length under maximum tension, Fracture energy: Stress-strain AUC. *P*< 0.05 vs. control group.

***Tensiometry***

The tissue stress and strain were increased both in incision and excision wounds compared to their control groups (*P*< 0.05) (Figures 3,4). 

## Discussion

The results of the present study show the incremental effects on wound healing percentage in treated groups with *Teucrium polium* honey compared to control ones (Table 1). Healing process in incision wounds was faster than the excision ones. 

Some reports have also shown the effects of wound phenotype (incision, excision) on the skin wound healing as the incision wound healing process had shorter time compared to excision ones (14,15). Zhang *et al* showed that in the excision wounds after wound recovery, the amount of scar formation was larger than the incision wounds. They also reported that wounds excision had disadvantages such as the difficulty in determining tissue vitality, bleeding and huge injury to normal tissues, and the damage to blood vessels and nerves (16). It was demonstrated that the wound incision might be superior to the wound excision for wound management (16). 

Plant products are widely used as medicaments for wound healing in many clinical situations. *Teucrium polium* used as an herb adopted for inflammatory conditions has white, pale cream flowers with several phenolic compounds, primarily the flavonoid family such as cirsimaritin, eupatorin, apigenin, cirsiliol and sterols. Beta-caryophyllene, beta-pinene, and alpha-pinene are the essential oil composition of the plant (17). High antioxidant activity of *Teucrium polium* honey has also been depicted in some studies (18,19) which was mainly due to the phenolic compounds such as hydroxybenzoic acid derivatives, caffeic acid, ferulic acid as well as flavonoid derivatives (10,20). Wound healing facilitation as we showed in the current study may be due to antioxidant, immunomodulating of sterol components or some other unknown activities.

 Previous studies also showed that honey had wound healing properties which can be ascribed to its antimicrobial activity. The antimicrobial activity can be effective against a broad spectrum of bacterial species especially those of medical importance (21). It has been again reported that a moist wound environment promotes healing and has a high viscosity that helps providing a protective barrier to prevent infection (22). Although the antimicrobial activity of honey has been effectively established against an extensive spectrum of microorganisms, it differs depending on the type of honey. *Teucrium polium* is widely used in inflammatory conditions, rheumatism and ulcers (10,11). Accordingly, *Teucrium polium* honey may have positive effects on wound healing process in comparison with control group in the present study.

We also showed that, tissue tensile strength, the force per unit of the cross-sectional area needed to break the wound; increased in rats receiving *Teucrium polium* honey in comparison to the control groups (Figures 3, 4). In stress-strain tests, the buildup force or stress is measured as the specimen deformation at a constant rate. In this test, the slope of the initial strength-line portion of the stress-strain curve is the elastic stiffness of the materials. In a tensile test, this stiffness is Young’s stiffness. The end of the curve denotes the failure of materials to transform load-deformity into stress-strain curves. Tensile strength is one of the most important factors in wound healing, and is a valuable measure that reflects the subdermal organization of the collagen fibers in the newly deposited collagen (19,23,24). Management of patients with full-thickness skin wound continues to challenge physicians and surgeons in area of cosmetic dermatologic surgery and some medications such as *Teucrium polium* honey may be used to accelerate the healing of full thickness skin wounds. As we noted in the histopathological part of our study, wound healing process was grossly improved in incision wounds post *Teucrium polium* honey application incomparison with excision ones. Drastic progression in the repair process, cellular recruitment and injured area were also seen in the treated group. Thus, *Teucrium polium* honey may have positive effects on the maturation, deposition, and correct orientation of collagen fibers at injured area which in return can increase tissue tensile strength.

## Conclusion

The present study demonstrates that *Teucriumpolium* honey can accelerate wound healing and tensile strength of the skin wound in rats.
